# Excess adiposity and early cardiometabolic risk markers among adolescents and young adults in 70 countries: a multi-country cross-sectional study

**DOI:** 10.1016/j.eclinm.2026.104282

**Published:** 2026-10-27

**Authors:** Setognal B. Aychiluhm, Vivian Isaac, Allen G. Ross, Subash Thapa, Kedir Y. Ahmed

**Affiliations:** a Rural Health Research Institute, Charles Sturt University, Orange, NSW, Australia; b Institute of Public Health, College of Medicine and Health Sciences, University of Gondar, Gondar, Ethiopia; c School of Allied Health, Exercise & Sports Sciences, Faculty of Science & Health, Charles Sturt University, Albury, NSW, Australia; d College of Medicine, Alfaisal University, Riyadh, Kingdom of Saudi Arabia; e Discipline of General Practice, School of Medicine, Adelaide University, Adelaide, SA, Australia; f Translational Health Research Institute, Western Sydney University, Sydney, NSW, Australia

**Keywords:** Excess adiposity, Cardiometabolic risk, Adolescents and young adults

## Abstract

**Background:**

The recent Lancet Commission recommended moving beyond body mass index (BMI)-only assessment by confirming excess adiposity with waist-based measures or, where available, direct body fat assessment. However, global evidence on the prevalence, classification, and early cardiometabolic relevance of anthropometrically confirmed excess adiposity (hereafter excess adiposity) among adolescents and young adults remains limited. We conducted this study across 70 countries in six World Health Organization (WHO) regions.

**Methods:**

In this cross-sectional study, we used nationally representative WHO STEPS data from 70 countries between 2010 and 2023. Excess adiposity was defined using a Lancet Commission-informed anthropometric framework incorporating BMI, waist circumference (WC), and waist-to-height ratio (WHtR). Random-effects meta-analysis estimated prevalence, and regression models examined associations with cardiometabolic risk markers.

**Findings:**

Among 60,634 individuals aged 15–29 years, the prevalence of excess adiposity was 33.7% (95% CI 29.4–38.0), ranging from 19.1% in South-East Asia to 48.2% in the Eastern Mediterranean region. Notably, 15.7% of participants had waist-confirmed adiposity, defined by both high WC and WHtR ≥ 0.50, despite not meeting BMI criteria for obesity. Higher odds of excess adiposity were observed in upper-middle-income countries (OR 2.54, 95% CI 1.40–4.60), high-income countries (OR 5.92, 95% CI 3.23–10.8), and physically inactive participants (OR 1.21, 95% CI 1.14–1.29). Excess adiposity was also associated with higher odds of having at least one (OR 2.15, 95% CI 1.86–2.49) and multiple (OR 4.09, 95% CI 3.20–5.23) cardiometabolic risk markers.

**Interpretation:**

This study found that nearly one-third of adolescents and young adults had excess adiposity. Notably, one in six participants had waist-confirmed adiposity despite not meeting BMI criteria for obesity, identifying a group with greater cardiometabolic risk burden that would be missed by BMI alone. These findings support the Lancet Commission’s move beyond BMI-only assessment and highlight the need to include waist-based measures in surveillance and prevention strategies to identify adiposity-related cardiometabolic risk earlier.

**Funding:**

None.

**Research in context**

**Evidence before this study**

We searched PubMed for studies published up to May 2026 using terms related to confirmed excess adiposity, central adiposity, waist-based measures, and cardiometabolic risk among adolescents and young adults. Existing evidence on confirmed excess adiposity remains limited and is largely based on adult populations or single-country studies. In younger populations, evidence suggests that central adiposity is associated with increased cardiometabolic risk, including among individuals without BMI-defined obesity, highlighting the limitations of BMI-based assessment alone. However, multinational evidence remains scarce on the prevalence, classification, associated factors, and early cardiometabolic risk of confirmed excess adiposity among adolescents and young adults.

**Added value of this study**

This study provides multinational evidence on confirmed excess adiposity among adolescents and young adults, using nationally representative data from 70 countries. Nearly one-third of participants met criteria for confirmed excess adiposity, with substantial variation across WHO regions and country income groups. Notably, one in six participants had waist-confirmed adiposity despite not meeting BMI criteria for obesity, identifying a group with higher early cardiometabolic risk that would be missed by BMI alone. These findings show the value of adding waist-based measures to BMI for identifying adiposity-related cardiometabolic risk in young populations.

**Implications of all the available evidence**

BMI alone may miss many adolescents and young adults with adiposity-related cardiometabolic risk. Our findings support the Lancet Commission’s move beyond BMI-only assessment and highlight the value of adding simple waist-based measures to obesity surveillance and prevention strategies. Earlier identification of excess adiposity and related risk could support more targeted prevention before cardiometabolic risk accumulates.

## Introduction

Body mass index (BMI) alone does not adequately capture body fat or early cardiometabolic risk, as it does not distinguish between fat and muscle or indicate where visceral fat is stored.^1^ People with a normal BMI but excess abdominal fat (“normal-weight obesity”) are often missed, while others with a high BMI but healthier fat distribution may be misclassified.^1^, ^2^

This misclassification may be more prominent in adolescents and young adults, delaying early preventive care and leading to increased cardiometabolic risk in adulthood and late life.^3^ Therefore, incorporating waist-based measurements improves risk assessment by more accurately identifying excess adiposity and detecting early cardiometabolic risk in individuals without overt disease, supporting earlier, more targeted, and proportionate interventions at both individual and population levels.^4^, ^5^

Recent evidence highlights the extent to which BMI alone can under-detect excess adiposity. In a U.S. study of more than 300,000 adults, obesity prevalence increased from 42.9% to 68.6% when waist-based measures were incorporated, identifying many people who would not have been classified as obese using BMI alone.^6^ Similarly, our previous multi-country study found that more than one in five adults with normal BMI had abdominal obesity, a phenotype associated with elevated cardiometabolic risk.^7^

To address these limitations, the Lancet Diabetes & Endocrinology Commission (2025) proposed a broader framework for obesity assessment that combines BMI with waist-based measures, including waist circumference (WC), waist-to-height ratio (WHtR), and waist-to-hip ratio (WHR), or with direct assessments of body fat, such as dual-energy X-ray absorptiometry (DXA).^4^ Crucially, the Commission distinguished clinical obesity, where excess adiposity is accompanied by organ dysfunction or functional limitation, from preclinical obesity, where excess adiposity is present without overt clinical dysfunction but with increased future health risk.^4^ This distinction is particularly relevant during adolescence and young adulthood, when excess adiposity and related cardiometabolic risk may become established and contribute to longer-term health trajectories.^8^

Cardiometabolic risk factors that emerge during childhood and adolescence can persist into adulthood and contribute to subsequent cardiovascular morbidity and mortality. The cumulative burden of cardiometabolic risk during these developmental stages has been associated with an increased risk of fatal and non-fatal cardiovascular events in adulthood, with adiposity-related risk trajectories becoming evident by late adolescence.^9^, ^10^, ^11^ Adolescence and young adulthood, therefore, represent critical windows for prevention, as risk trajectories established during these periods may influence cardiovascular health across the life course.

Previous studies showed that visceral fat gained in late adolescence and early adulthood predicts later hypertension, dyslipidaemia, type 2 diabetes, and metabolic syndrome, even among individuals with a normal BMI.^12^, ^13^ Reliance on BMI alone therefore obscures this early, waist-centred risk and leads to missed opportunities for prevention. Incorporating waist-based measures enables the identification of central adiposity, allowing timely, targeted interventions that can alter long-term cardiometabolic trajectories and reduce progression to clinical obesity and chronic disease.^14^

Despite this, evidence among adolescents and young adults remains scarce, particularly from multi-country studies covering diverse WHO regions and country income groups. This gap limits understanding of how common excess adiposity is in young populations, how it is classified by BMI and waist measures, and whether it is already linked to cardiometabolic risk. Addressing this gap is important for improving early prevention and strengthening global adiposity surveillance. This study therefore aimed to: (i) estimate the prevalence of excess adiposity among adolescents and young adults; (ii) assess variation by WHO region and country income group; (iii) describe BMI–waist adiposity patterns; (iv) identify key determinants; and (v) examine associations with cardiometabolic risk markers, including hypertension, diabetes, and high total cholesterol.

## Methods

### Data sources

This study used data from the World Health Organization (WHO) STEPwise approach to non-communicable disease (NCD) risk factor surveillance (STEPS), a standardised, population-based framework for monitoring key behavioural and metabolic risk factors for non-communicable diseases.^15^ The STEPS survey comprises three modules: Step 1 collects socio-demographic information and self-reported behavioural risks (e.g., alcohol consumption, diet, and physical activity); Step 2 involves physical measurements (e.g., height, weight, waist circumference, and blood pressure); and Step 3 includes biochemical tests (e.g., fasting blood glucose and cholesterol). These population-based, cross-sectional household surveys typically use multistage cluster sampling with weights to ensure nationally representative data.^15^ Within each selected household, one eligible member was randomly selected for interview using the STEPS electronic data-collection application, which lists all eligible household members and performs the selection electronically.^15^ Each country sets the minimum eligibility age for participant selection in advance of fieldwork.

### Study population

The analysis included non-pregnant adolescents and young adults aged 15–29 years from 70 nationally representative WHO STEPS surveys conducted between 2010 and 2023 (N = 60,634), covering all six WHO regions: Africa, the Americas, the Eastern Mediterranean, Europe, South-East Asia, and the Western Pacific. Sub-national surveys restricted to specific provinces or cities were excluded during dataset construction. The derivation of the analytic sample is shown in Supplementary Fig. S1. This study adhered to the Strengthening the Reporting of Observational Studies in Epidemiology (STROBE) guidelines for cross-sectional studies.^16^

### Variables and measurements

#### Confirmed excess adiposity

The primary outcome was anthropometrically confirmed excess adiposity, operationalised using a Lancet Commission-informed anthropometric framework.^4^ Participants were classified as having confirmed excess adiposity if they met any of the following criteria: (i) elevated BMI (e.g., BMI ≥30 kg/m^2^) combined with at least one elevated waist-based measure (e.g., WC ≥ 94 cm for men, ≥80 cm for women, or WHtR ≥0.5); (ii) elevated values for both WC and WHtR; or (iii) extreme BMI alone (BMI ≥40 kg/m^2^). Sex, age, and ethnicity-specific cutoffs were applied to account for variations in body composition and cardiometabolic risk. For adolescents, BMI-for-age z-scores were calculated using the World Health Organization 2007 growth reference standards and classified using standard WHO cut-points. For ages 15–17 years, obesity-range BMI was defined as BAZ ≥+2 SD and very-high BMI as BAZ ≥+3 SD.^17^ For ages 18–29 years, obesity-range BMI was defined as BMI ≥27.5 kg/m^2^ in Asian participants and ≥30.0 kg/m^2^ in non-Asian participants; very-high BMI was defined as ≥37.5 kg/m^2^ and ≥40.0 kg/m^2^, respectively. High waist circumference was defined as ≥90 cm in Asian males and ≥80 cm in Asian females, and ≥94 cm in non-Asian males and ≥80 cm in non-Asian females. WHtR ≥0.50 was used as the second waist-based marker (Supplementary Table S1). Because WHO STEPS do not comprehensively assess organ dysfunction, symptoms, functional limitation, or clinical history, this study estimated anthropometrically confirmed excess adiposity and its association with early cardiometabolic risk, rather than diagnosing clinical or preclinical obesity.

#### Cardiometabolic risk markers

The secondary outcomes were three cardiometabolic risk markers: hypertension, diabetes, and high total cholesterol. Three readings of systolic blood pressure (SBP) and diastolic blood pressure (DBP) were taken for each participant, with a 3-min rest provided between measurements. For analysis, the mean of the second and third readings was used. Hypertension was defined as systolic blood pressure (SBP) ≥140 mmHg, and/or a diastolic blood pressure (DBP) ≥90 mmHg, or current use of antihypertensive medication.^18^ Diabetes was defined as a fasting blood glucose (FBG) ≥7 mmol/L (≥126 mg/dL), or the use of glucose-lowering medication.^19^ High total cholesterol was defined as total cholesterol ≥5.0 mmol/L (≥190 mg/dL) or self-reported use of lipid-lowering medication^15^[Supplementary Table S2].

Cardiometabolic burden was assessed by classifying participants according to the number of these three risk markers present: none, one, or two or more. A cardiometabolic risk-marker count (range 0–3) was calculated as the sum of hypertension, diabetes, and elevated total cholesterol among participants with non-missing information on all three markers. Two binary composite outcomes were derived from this count: any risk marker (count ≥1) and multiple risk markers (count ≥2). Participants missing information on any of the three markers were excluded from analyses based on the composite risk-marker count.

#### Explanatory variables

Explanatory variables included demographic factors (age group and sex) and socioeconomic factors (educational status, occupation, and country income group). Lifestyle factors encompassed physical activity, sedentary behaviour, fruit and vegetable intake, smoking, alcohol consumption, and consuming processed high-salt foods (Supplementary Table S3).

### Data management

Data cleaning, coding, and management were performed using Stata MP (version 18; StataCorp, College Station, TX, USA). After cleaning, the dataset was imported into R (version 4.5.1) for further statistical analysis. Biochemical measurements, including total cholesterol and fasting blood glucose, were reported in milligrams per deciliter (mg/dL) in some countries and millimoles per liter (mmol/L) in others; all values were harmonised to mg/dL using standard conversion factors (fasting blood glucose: mg/dL = mmol/L × 18.02; total cholesterol: mg/dL = mmol/L × 38.67). In STEPS surveys, biochemical measurements may be obtained using point-of-care dry-chemistry devices (e.g., CardioChek PA, Cholestech LDX) with capillary blood samples, or using wet-chemistry methods with venous blood samples analysed at a central laboratory. The specific approach varies by country. No additional retrospective calibration or country-specific correction factors were applied in the pooled analysis. To ensure data quality and analytical reliability, individuals with missing values for essential anthropometric indicators (weight, height, waist circumference) or key clinical measures (blood pressure, total cholesterol, and diabetes status) were excluded. Additionally, individuals with implausible values were removed based on established global thresholds (e.g., systolic blood pressure <70 or >250 mmHg).

### Statistical analysis and modelling

The pooled global prevalence of excess adiposity was estimated using a random-effects meta-analysis that combined country-specific prevalence estimates while accounting for between-country heterogeneity. Between-country variance (τ^2^) was estimated using Restricted Maximum Likelihood (REML), and confidence intervals were adjusted using the Hartung-Knapp method to improve accuracy. Heterogeneity was quantified using the I^2^ statistic and Cochran’s Q test. In addition to the global pooled estimate, subgroup meta-analyses were conducted by WHO region and by World Bank income group (low, lower-middle, upper-middle, and high-income countries). These analyses allowed assessment of geographic and economic variation in the burden of excess adiposity. Forest plots were generated to display country- and subgroup-specific estimates alongside the pooled prevalence.

Multilevel logistic regression was used to identify factors associated with excess adiposity in the pooled global sample, with country included as a random effect. Region-specific associations were estimated using survey-weighted logistic regression within each WHO region. Separate survey-weighted logistic regression models examined the association between excess adiposity and cardiometabolic burden, adjusting for covariates. Multicollinearity was assessed using the generalised variance inflation factor (GVIF), with a cutoff of 5; no multicollinearity was detected. All models accounted for survey weights and stratification by country-year survey; primary sampling unit identifiers were not consistently available across the harmonised dataset, and each observation was treated as its own primary sampling unit. Results are presented as odds ratios (ORs) with 95% confidence intervals (CIs).

### Ethics

This study used de-identified, secondary data obtained through the WHO NCD Microdata Repository. Approval for this secondary analysis was granted by the Human Research Ethics Committee at Charles Sturt University (H25075). Each original national survey was conducted under its own country-specific ethical approval and informed consent procedures; as participants could not be identified from the data used here, separate consent for this analysis was not required.

### Role of the funding source

This study received no funding.

## Results

### Description of study participants

A total of 60,634 adolescents and young adults aged 15–29 years from 70 countries were included. Nearly half were from Africa (51.1%), followed by the Western Pacific (14.8%), the Eastern Mediterranean (10%), South-East Asia (9.0%), Europe (8.2%), and the Americas (6.9%). The median age was 24 years (IQR: 20–27), with nearly half aged 18–24 years (49.4%), and males comprised just over half of the sample (51.1%) [Table 1].

**Table 1:** Participant characteristics by WHO region.

Characteristic	Africa (n = 31,022)	Region of the Americas (n = 4161)	Eastern Mediterranean (n = 6083)	Europe (n = 4956)	South-East Asia (n = 5458)	Western Pacific (n = 8954)	Global (n = 60,634)
Age group							
15–17 years	2168 (7.0%)	0 (0.0%)	27 (0.4%)	0 (0.0%)	241 (4.4%)	311 (3.5%)	2747 (4.5%)
18–24 years	14,708 (47.4%)	2263 (54.4%)	3786 (62.2%)	2461 (49.7%)	2628 (48.1%)	4117 (46.0%)	29,963 (49.4%)
25–29 years	14,146 (45.6%)	1898 (45.6%)	2270 (37.3%)	2495 (50.3%)	2589 (47.4%)	4526 (50.5%)	27,925 (46.1%)
Sex							
Males	15,782 (50.9%)	2100 (50.5%)	3360 (55.2%)	2602 (52.5%)	2817 (51.6%)	4342 (48.5%)	31,002 (51.1%)
Females	15,240 (49.1%)	2061 (49.5%)	2723 (44.8%)	2354 (47.5%)	2641 (48.4%)	4612 (51.5%)	29,632 (48.9%)
Education							
Secondary or more	11,528 (38.8%)	2425 (61.2%)	2746 (49.1%)	3051 (86.6%)	2680 (51.1%)	6200 (70.4%)	28,631 (50.4%)
No formal schooling	4079 (13.7%)	75 (1.9%)	757 (13.5%)	12 (0.3%)	384 (7.3%)	306 (3.5%)	5614 (9.9%)
Primary or less	14,085 (47.4%)	1460 (36.9%)	2088 (37.3%)	460 (13.1%)	2184 (41.6%)	2307 (26.2%)	22,584 (39.7%)
Occupation							
Working	19,632 (64.5%)	3210 (80.5%)	3949 (65.4%)	2125 (54.4%)	3798 (70.1%)	6327 (74.1%)	39,042 (66.9%)
Not working	10,799 (35.5%)	778 (19.5%)	2087 (34.6%)	1785 (45.6%)	1622 (29.9%)	2206 (25.9%)	19,277 (33.1%)
Country income group							
Low-income	17,082 (55.1%)	0 (0.0%)	1827 (30.0%)	0 (0.0%)	0 (0.0%)	0 (0.0%)	18,909 (31.2%)
Lower-middle income	10,010 (32.3%)	1041 (25.0%)	1571 (25.8%)	1040 (21.0%)	5458 (100.0%)	2836 (31.7%)	21,956 (36.2%)
Upper-middle income	3930 (12.7%)	1143 (27.5%)	951 (15.6%)	3916 (79.0%)	0 (0.0%)	3199 (35.7%)	13,139 (21.7%)
High-income	0 (0.0%)	1977 (47.5%)	1734 (28.5%)	0 (0.0%)	0 (0.0%)	2919 (32.6%)	6630 (10.9%)
Physical activity							
Active	25,415 (88.8%)	3070 (78.1%)	3764 (63.6%)	4172 (86.7%)	4497 (85.0%)	6595 (76.7%)	47,512 (83.1%)
Inactive	3206 (11.2%)	861 (21.9%)	2157 (36.4%)	639 (13.3%)	796 (15.0%)	2002 (23.3%)	9660 (16.9%)
High sedentary time							
No	27,148 (92.0%)	3428 (88.0%)	4625 (78.1%)	4482 (91.3%)	4976 (92.4%)	7891 (89.5%)	52,550 (89.9%)
Yes	2375 (8.0%)	468 (12.0%)	1299 (21.9%)	425 (8.7%)	412 (7.6%)	922 (10.5%)	5901 (10.1%)
Smoking							
No	28,200 (91.7%)	3459 (84.2%)	4027 (72.2%)	3911 (78.9%)	4485 (82.2%)	5722 (68.2%)	49,803 (84.1%)
Yes	2537 (8.3%)	651 (15.8%)	1548 (27.8%)	1045 (21.1%)	973 (17.8%)	2670 (31.8%)	9424 (15.9%)
Alcohol consumption							
Non-drinker	19,711 (80.5%)	1614 (55.1%)	4353 (98.8%)	2816 (75.2%)	4417 (91.9%)	4474 (75.4%)	37,384 (80.7%)
Drinker	4772 (19.5%)	1315 (44.9%)	53 (1.2%)	929 (24.8%)	388 (8.1%)	1463 (24.6%)	8919 (19.3%)
Fruit and vegetable servings							
Adequate (≥5/day)	6432 (29.5%)	928 (25.3%)	1277 (23.8%)	2580 (53.8%)	1484 (36.0%)	2423 (34.8%)	15,124 (32.4%)
Inadequate (<5/day)	15,365 (70.5%)	2735 (74.7%)	4085 (76.2%)	2219 (46.2%)	2634 (64.0%)	4535 (65.2%)	31,573 (67.6%)
Processed foods high in salt							
Infrequent	11,816 (58.1%)	1579 (41.9%)	1548 (36.0%)	1853 (40.0%)	1612 (34.3%)	1741 (31.8%)	20,150 (46.6%)
Regular consumption	8518 (41.9%)	2190 (58.1%)	2750 (64.0%)	2776 (60.0%)	3087 (65.7%)	3730 (68.2%)	23,050 (53.4%)

Note: Values are weighted n (%). Totals may vary slightly because of missing data.

Globally, physical inactivity was found in 16.9% of young people, was highest in the Eastern Mediterranean (36.4%) and lowest in Africa (11.2%). Only one-third of young people (32.4%) met the WHO recommendation of consuming at least 5 servings of fruits and vegetables daily, with most regions below 50%. Additionally, regular consumption of processed food high in salt was very common, reported by nearly 53.4% of all participants and over 50% in most regions [Table 1]. Additional country-level descriptions of the main outcomes are presented in Supplementary Table S4.

### Prevalence of excess adiposity

The overall pooled prevalence of excess adiposity among young people aged 15–29 years was 33.7% (95% CI, 29.4%–38.0%), with variations across countries (I^2^ = 99%, τ² = 0.0316) [Fig. 1]. Country-level prevalence ranged from 6.6% in Ethiopia to 77.2% in Tonga and 77.1% in the Cook Islands. Prevalence varied across WHO regions, with the highest in the Eastern Mediterranean (48.2%; 95% CI, 40.1%–56.3%) and Western Pacific (47.7%; 95% CI, 36.5%–58.9%) and lowest in Africa (21.2%; 95% CI, 17.4%–24.9%) and South-East Asia (19.1%; 95% CI, 8.3%–29.9%) [Fig. 2]. Subgroup differences by country income group were significant, with prevalence increasing from 21.6% (95% CI 12.3–30.9) in low-income countries to 51.7% (95% CI 44.0–59.4) in high-income countries [Fig. 3].

**Fig. 1:**
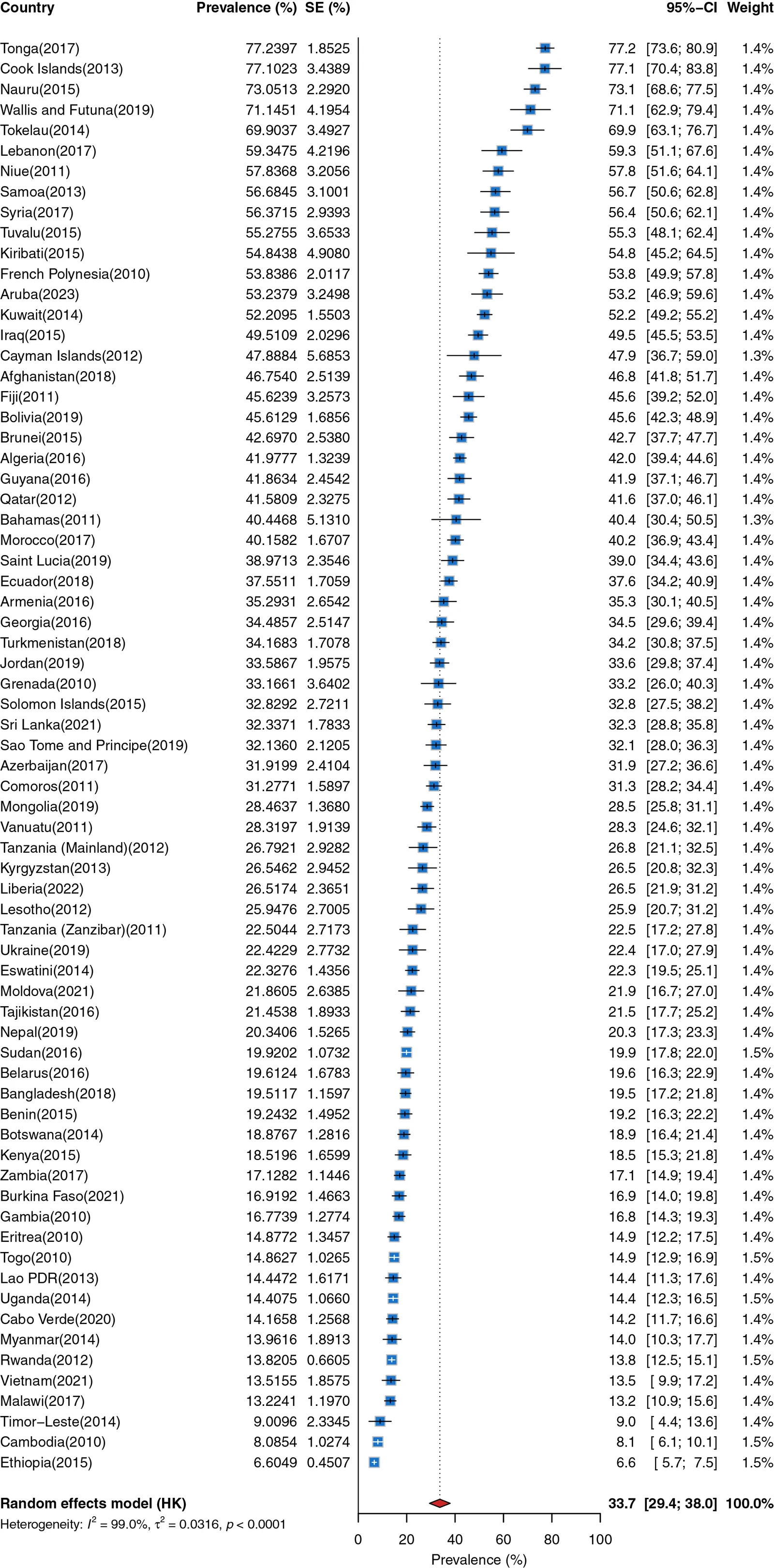
Pooled prevalence of confirmed excess adiposity. Country-specific estimates are shown with 95% CIs. The diamond shows the pooled random-effects estimate.

**Fig. 2:**
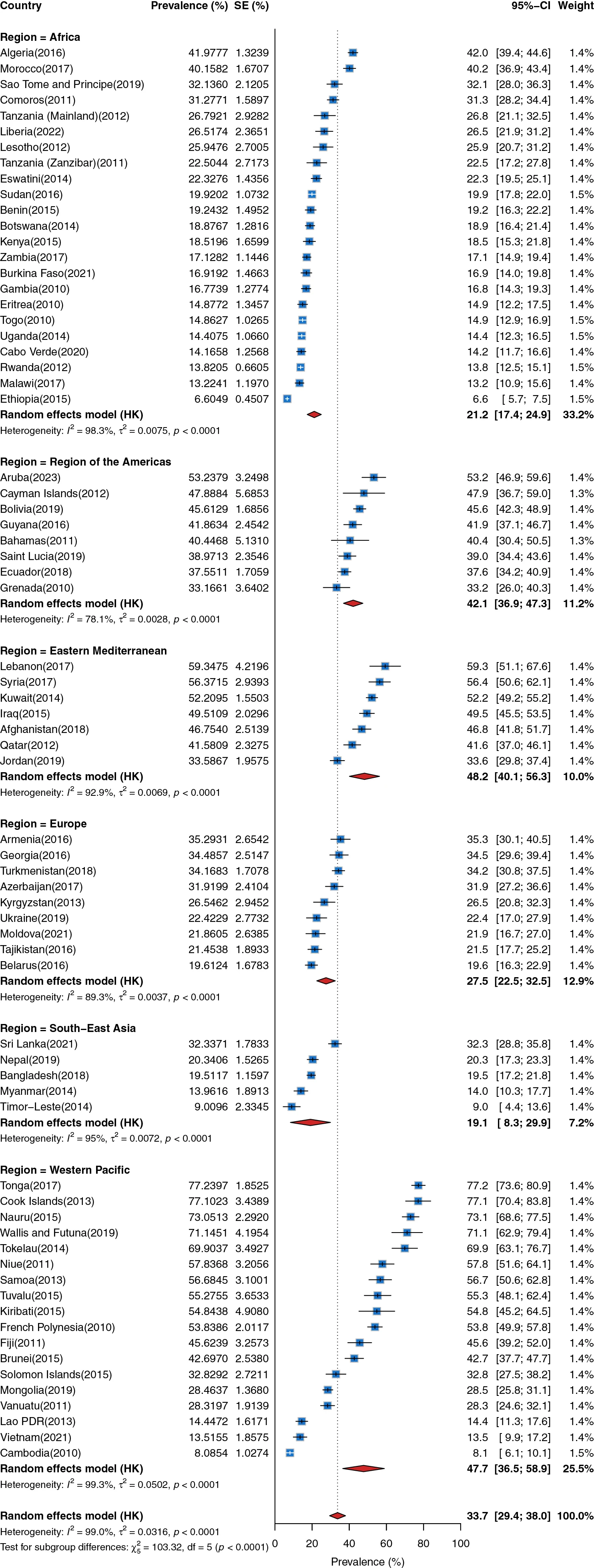
Confirmed excess adiposity by WHO region. Country-specific estimates are grouped by WHO region. Diamonds show regional and overall pooled random-effects estimates.

**Fig. 3:**
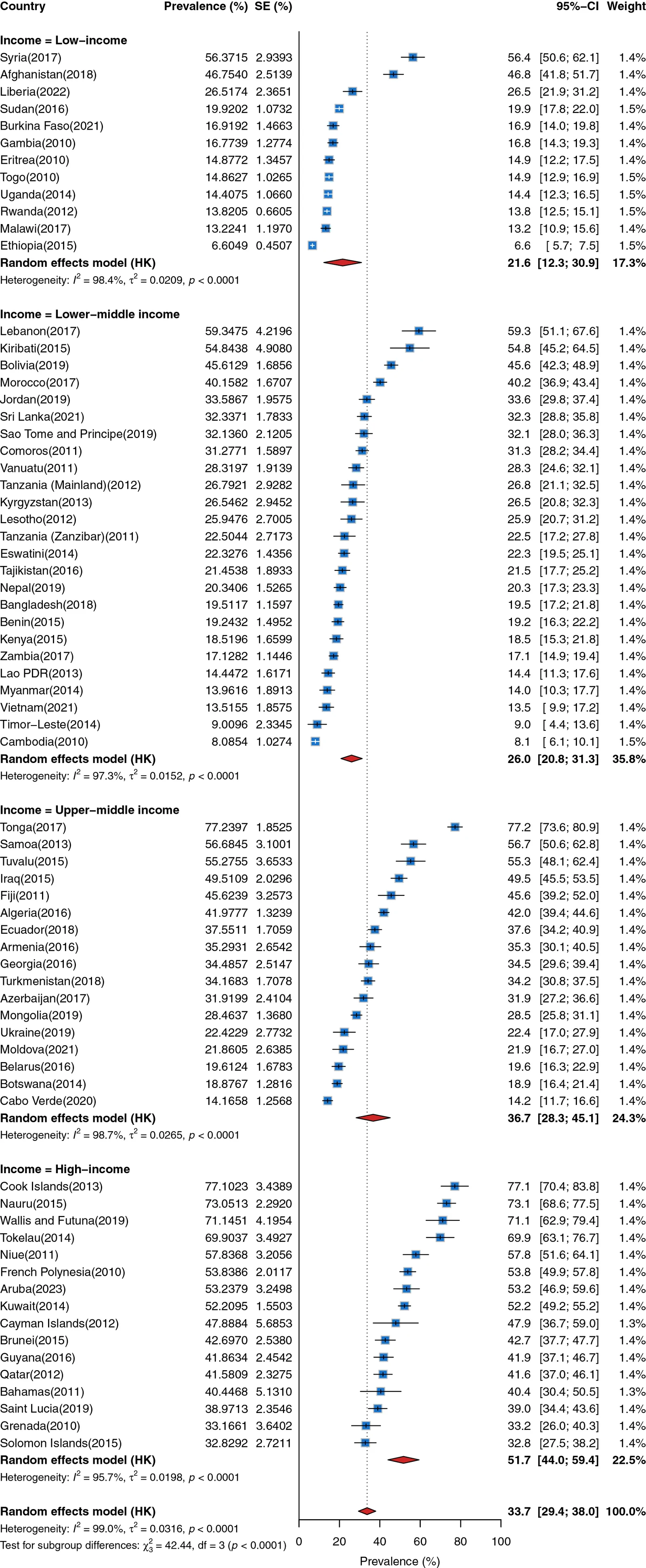
Confirmed excess adiposity by World Bank income group. Diamonds show regional and overall pooled random-effects estimates.

### BMI–waist-based classification of excess adiposity

Among all participants, 12.7% had BMI-defined obesity, 38.3% met at least one waist criterion, and 26.6% met both high WC and WHtR ≥ 0.50 [Table 2]. In the mutually exclusive BMI–waist classification, BMI-only obesity was uncommon (0.8%), whereas 15.7% had waist-confirmed adiposity without BMI-defined obesity, and 11.9% had BMI-plus-waist or very-high BMI adiposity [Supplementary Fig. S2]. Confirmed excess adiposity was two- to three-fold more prevalent in females than males across all age groups, with the absolute sex difference widening from adolescence to young adulthood [Table 2].

**Table 2:** Cardiometabolic risk burden across BMI–waist adiposity categories.

Classification	No risk markers	1 risk marker	≥2 risk markers	Any risk marker OR (95% CI)	Multiple risk markers OR (95% CI)
BMI-waist adiposity					
No adiposity evidence	22,763 (80.9)	4942 (17.6)	417 (1.5)	Ref.	Ref.
One Waist criterion only	3628 (72.2)	1234 (24.5)	165 (3.3)	1.55 (1.33–1.79)	2.26 (1.59–3.22)
Waist-confirmed excess adiposity	4720 (69.1)	1800 (26.3)	313 (4.6)	1.92 (1.64–2.25)	3.93 (2.80–5.52)
BMI-plus-waist or very-high BMI adiposity	2654 (56.6)	1587 (33.8)	449 (9.6)	3.08 (2.58–3.68)	6.89 (5.11–9.28)
Excess adiposity					
No excess adiposity	26,555 (79.5)	6242 (18.7)	625 (1.9)	Ref.	Ref.
Excess adiposity	7374 (64.0)	3386 (29.4)	761 (6.6)	2.15 (1.86–2.49)	4.09 (3.20–5.23)

Note: BMI = body mass index; OR = adjusted odds ratio; CI = confidence interval; Ref. = reference category. Cardiometabolic risk markers were hypertension, diabetes, and elevated total cholesterol. Adjusted models were fitted using survey-weighted logistic regression and adjusted for age group, sex, education, occupation, country income, physical activity, high sedentary time, and smoking. BMI-only obesity category was excluded from cardiometabolic outcome analyses because of sparse cell counts.

### Factors associated with excess adiposity

Excess adiposity was strongly associated with older age, female sex, higher country income, and physical inactivity. Compared with adolescents aged 15–17 years, the odds increased among those aged 18–24 years (OR 1.96, 95% CI 1.66–2.31) and were almost four times higher among those aged 25–29 years (OR 3.83, 95% CI 3.25–4.52). Females had substantially higher odds than males globally (OR 4.06, 95% CI 3.86–4.28), with the strongest association observed in Africa (OR 9.27, 95% CI 8.13–10.6). Higher odds were also observed in upper-middle-income countries (OR 2.54, 95% CI 1.40–4.60) and high-income countries (OR 5.92, 95% CI 3.23–10.8). Physical inactivity was associated with excess adiposity globally (OR 1.21, 95% CI 1.14–1.29), with stronger associations observed in South-East Asia (OR 1.74, 95% CI 1.32–2.29) and the Western Pacific (OR 1.54, 95% CI 1.30–1.83) [Table 3]. Age- and sex-stratified determinants are presented in Supplementary Table S5.

**Table 3:** BMI-Waist based excess adiposity classification by sex and age group.

Classification	Male			Female			Total 60,634
	15–17 years 1485 (2.4%)	18–24 years 15,824 (26.1%)	25–29 years 13,694 (22.6%)	15–17 years 1262 (2.1%)	18–24 years 14,139 (23.3%)	25–29 years 14,231 (23.5%)	
BMI defined obesity							
No	1412 (95.1%)	14,301 (90.4%)	11,973 (87.5%)	1201 (95.1%)	12,266 (86.8%)	11,740 (82.6%)	52,892 (87.3%)
Yes	73 (4.9%)	1511 (9.6%)	1707 (12.5%)	62 (4.9%)	1861 (13.2%)	2474 (17.4%)	7688 (12.7%)
Very-high BMI							
No	1433 (96.5%)	15,598 (98.6%)	13,465 (98.4%)	1229 (97.4%)	13,853 (98.1%)	13,857 (97.5%)	59,434 (98.1%)
Yes	51 (3.5%)	214 (1.4%)	215 (1.6%)	33 (2.6%)	274 (1.9%)	357 (2.5%)	1146 (1.9%)
At least one waist criterion							
No	1344 (92.7%)	11,682 (73.8%)	8962 (65.5%)	954 (77.2%)	7775 (55.0%)	6654 (46.8%)	37,371 (61.7%)
Yes	105 (7.3%)	4140 (26.2%)	4727 (34.5%)	281 (22.8%)	6361 (45.0%)	7574 (53.2%)	23,189 (38.3%)
Both waist criteria							
No	1418 (97.8%)	13,657 (86.3%)	11,207 (81.9%)	1062 (86.0%)	9131 (64.6%)	7998 (56.2%)	44,473 (73.4%)
Yes	32 (2.2%)	2164 (13.7%)	2483 (18.1%)	173 (14.0%)	5004 (35.4%)	6231 (43.8%)	16,087 (26.6%)
BMI-plus-waist or very-high BMI							
No	1417 (95.4%)	14,399 (91.1%)	12,113 (88.5%)	1206 (95.5%)	12,355 (87.5%)	11,881 (83.6%)	53,370 (88.1%)
Yes	68 (4.6%)	1414 (8.9%)	1567 (11.5%)	57 (4.5%)	1772 (12.5%)	2333 (16.4%)	7210 (11.9%)
Confirmed excess adiposity							
No	1408 (94.9%)	13,426 (84.9%)	10,978 (80.2%)	1058 (83.8%)	9064 (64.2%)	7919 (55.7%)	43,853 (72.4%)
Yes	76 (5.1%)	2386 (15.1%)	2702 (19.8%)	204 (16.2%)	5063 (35.8%)	6295 (44.3%)	16,727 (27.6%)

Note: Values are weighted n (%); categories are not mutually exclusive. Confirmed excess adiposity comprises waist-confirmed adiposity and BMI-plus-waist or very-high BMI adiposity, defined using age- and ethnicity-specific BMI, waist circumference (WC), and waist-to-height ratio (WHtR) cut-offs. The participant-level prevalence of confirmed excess adiposity may differ from the pooled prevalence (Fig. 1), as the random-effects model accounts for between-country heterogeneity and weights country-specific estimates. BMI = body mass index. Totals may differ slightly due to rounding.

### Cardiometabolic risk burden

Compared with participants with no evidence of adiposity, waist-confirmed adiposity was associated with higher odds of any cardiometabolic risk marker (OR 1.92, 95% CI 1.64–2.25) and multiple risk markers (OR 3.93, 95% CI 2.80–5.52). The strongest associations were observed for BMI-plus-waist or very-high BMI adiposity, with ORs of 3.08 (95% CI, 2.58–3.68) for any risk marker and 6.89 (95% CI, 5.11–9.28) for multiple risk markers. Overall, confirmed excess adiposity was associated with a greater cardiometabolic burden, with ORs of 2.15 (95% CI, 1.86–2.49) for any risk marker and 4.09 (95% CI, 3.20–5.23) for multiple risk markers [Table 4]. The prevalence of individual cardiometabolic risk markers by age group is presented in Supplementary Table S6, and by BMI-waist adiposity category in Supplementary Table S7.

**Table 4:** Multivariable regression analysis of factors associated with excess adiposity across WHO regions.

Variable	Africa (N = 25,685)	Americas (N = 2804)	Eastern Mediterranean (N = 4287)	Europe (N = 4357)	South-East Asia (N = 4195)	Western Pacific (N = 7360)	Global (N = 49,407)
Age group							
15–17 years	Ref.	–	Ref.	–	Ref.	Ref.	Ref.
18–24 years	2.55 (2.02, 3.21)	Ref.	1.05 (0.20, 5.59)	Ref.	1.16 (0.66, 2.04)	5.16 (3.17, 8.40)	1.96 (1.66, 2.31)
25–29 years	4.16 (3.30, 5.24)	2.21 (1.82, 2.69)	1.88 (0.35, 10.0)	1.53 (1.30, 1.81)	2.95 (1.66, 5.21)	8.98 (5.50, 14.7)	3.83 (3.25, 4.52)
Sex							
Males	Ref.	Ref.	Ref.	Ref.	Ref.	Ref.	Ref.
Females	9.27 (8.13, 10.6)	4.03 (3.26, 4.98)	1.89 (1.50, 2.39)	1.41 (1.19, 1.67)	2.30 (1.71, 3.10)	2.73 (2.38, 3.13)	4.06 (3.86, 4.28)
Education							
Secondary or more	Ref.	Ref.	Ref.	–	Ref.	Ref.	Ref.
No formal schooling	0.87 (0.76, 0.99)	1.86 (1.02, 3.40)	1.81 (1.26, 2.61)	–	0.36 (0.21, 0.62)	0.41 (0.26, 0.65)	1.04 (0.95, 1.14)
Primary or less	1.03 (0.93, 1.15)	1.06 (0.84, 1.35)	0.95 (0.74, 1.21)	–	0.83 (0.64, 1.07)	0.84 (0.71, 0.99)	0.99 (0.93, 1.05)
Occupation							
Working	Ref.	Ref.	Ref.	–	Ref.	Ref.	Ref.
Not working	0.89 (0.81, 0.97)	0.91 (0.71, 1.17)	1.33 (1.05, 1.69)	–	1.25 (0.89, 1.77)	1.20 (1.03, 1.40)	1.11 (1.05, 1.17)
Country income group							
Low-income	Ref.	–	Ref.	–	–	–	Ref.
Lower-middle income	1.93 (1.76, 2.13)	Ref.	0.69 (0.51, 0.94)	Ref.	–	Ref.	1.23 (0.72, 2.13)
Upper-middle income	2.77 (2.41, 3.19)	0.75 (0.57, 1.00)	1.16 (0.88, 1.52)	1.43 (1.17, 1.76)	–	3.90 (3.10, 4.90)	2.54 (1.40, 4.60)
High-income	–	0.90 (0.67, 1.20)	1.47 (1.09, 1.98)	–	–	5.97 (4.79, 7.44)	5.92 (3.23, 10.8)
Physical activity							
Active	Ref.	Ref.	Ref.	Ref.	Ref.	Ref.	Ref.
Inactive	1.39 (1.24, 1.56)	1.22 (0.96, 1.57)	1.17 (0.96, 1.43)	1.31 (1.04, 1.63)	1.74 (1.32, 2.29)	1.54 (1.30, 1.83)	1.21 (1.14, 1.29)
High sedentary time							
No	Ref.	Ref.	Ref.	–	Ref.	Ref.	Ref.
Yes	1.30 (1.13, 1.51)	0.93 (0.69, 1.26)	1.02 (0.80, 1.29)	–	0.96 (0.67, 1.38)	1.07 (0.89, 1.28)	1.06 (0.98, 1.14)
Smoking							
No	Ref.	Ref.	Ref.	Ref.	Ref.	Ref.	Ref.
Yes	0.95 (0.75, 1.20)	0.86 (0.63, 1.17)	1.12 (0.87, 1.44)	1.06 (0.84, 1.33)	0.66 (0.47, 0.93)	1.24 (1.05, 1.46)	1.01 (0.94, 1.08)
Processed food high in salt							
Infrequent	–	Ref.	–	Ref.	Ref.	–	–
Regular consumption	–	1.06 (0.87, 1.29)	–	1.01 (0.86, 1.20)	0.84 (0.63, 1.12)	–	–
Fruit and vegetable serving							
Adequate (≥5/day)	–	Ref.	Ref.	Ref.	–	–	–
Inadequate (<5/day)	–	1.13 (0.89, 1.42)	0.97 (0.78, 1.20)	0.80 (0.68, 0.94)	–	–	–

Note. Ref. indicates the reference category. – indicates that the variable/category was not included in the respective model because data were unavailable, not applicable, or insufficient for estimation. Regional covariates were selected based on data availability and acceptable missingness within each WHO region. Models were adjusted for age group, sex, education, occupation, country income group, physical activity, high sedentary time, smoking, processed food high in salt, and fruit/vegetable servings, as available by region. Reference categories represent the lowest or baseline level of each variable. OR = odds ratio; CI = confidence interval; WHO = World Health Organization.

## Discussion

In this multi-country study of adolescents and young adults aged 15–29 years, almost one-third had confirmed excess adiposity when BMI and waist-based measures were considered together. The burden differed across WHO regions and country income groups, with higher prevalence in upper-middle-income and high-income settings. Excess adiposity was also more common with increasing age, among females, and among physically inactive participants.

Importantly, one in six participants had waist-confirmed adiposity despite not meeting BMI criteria for obesity. This group also showed higher cardiometabolic risk; they had higher odds of any cardiometabolic risk marker and almost four times the odds of multiple risk markers.

These findings suggest that BMI alone can misclassify adiposity-related risk and under-detect excess adiposity in young populations, supporting the inclusion of waist-based measures for earlier risk identification and prevention.

The recent Lancet Diabetes & Endocrinology Commission recommended moving beyond BMI-only assessment in obesity classification. It stated that BMI should be used mainly for screening or epidemiological purposes, and that excess adiposity should be confirmed using direct body-fat assessment where available, or anthropometric measures such as waist circumference, waist-to-height ratio, or waist-to-hip ratio. The Commission also distinguished clinical obesity, where excess adiposity is accompanied by organ or tissue dysfunction or functional limitation, from preclinical obesity, where excess adiposity is present without overt clinical dysfunction but with increased future health risk. This framework shows why BMI alone may miss central adiposity and early cardiometabolic risk.^4^

Our findings support moving beyond BMI-only assessment at the population level. Nearly one in six participants had waist-confirmed adiposity despite not meeting BMI criteria for obesity. This group also had greater cardiometabolic risk, including higher odds of any risk marker and almost four times the odds of multiple risk markers. Evidence from the All of Us Research Program similarly showed that individuals with anthropometric-only obesity, who would not be classified as obese using BMI thresholds alone, had higher risks of diabetes, cardiovascular disease, and mortality than those without obesity.^6^ These findings suggest that BMI alone can under-detect excess adiposity and misclassify adiposity-related risk in adolescents and young adults. This is consistent with previous studies showing that central fat accumulation, even at lower BMI levels, is associated with early cardiometabolic risk.^7^, ^20^

Our findings also revealed marked disparities in excess adiposity across regions and income groups by country. Prevalence was highest in the Eastern Mediterranean and Western Pacific and other high-income regions, consistent with evidence linking advanced nutrition transitions, widespread ultra-processed foods, and sedentary behaviour to rising adiposity.^7^, ^21^ In contrast, African and other low-income countries showed lower prevalence, reflecting earlier nutrition transitions, more minimally processed diets, higher physical activity, lower urbanisation, and limited access to energy-dense foods.^22^, ^23^ However, data from several low- and middle-income countries (LMICs) indicate a rapid increase in adiposity among adolescents and young adults, driven by greater consumption of energy-dense foods and declining physical activity.^24^, ^25^ These patterns suggest that while high- and upper-middle-income countries currently bear the greatest burden, many LMICs are experiencing a swift rise.

This same transition may also explain the regional variation observed in education. In Africa, South-East Asia, and the Western Pacific, no formal schooling was protective against excess adiposity, whereas in the Eastern Mediterranean region and the Region of the Americas it was associated with higher odds. This divergence aligns with the “reversal hypothesis” described in the global obesity literature: in less economically developed settings, lower education is linked to physically demanding labour and reduced access to energy-dense food, and is therefore protective, whereas in more economically transitioned settings the pattern reverses as lower-education groups gain greater exposure to inexpensive, energy-dense food while higher-education groups gain access to healthier alternatives.^26^, ^27^

The presence of cardiometabolic risk markers in adolescents and young adults highlights a critical window for prevention. These findings support adding waist-based measures and early metabolic risk markers to BMI-based assessment. Confirmed excess adiposity may help identify young people at higher cardiometabolic risk at an earlier stage, strengthening obesity and NCD surveillance in line with the WHO Global Action Plan for NCD prevention. The WHO STEPS currently do not capture all information required to distinguish clinical from preclinical obesity under the Lancet Commission’s framework, particularly organ dysfunction, symptoms, functional limitations, and clinical history. Future revisions of the WHO STEPS protocol or complementary modules could incorporate brief, validated measures of functional limitations and organ-specific symptoms to strengthen population-level surveillance of clinical and preclinical obesity.

This study has several strengths. It used nationally representative data across 70 countries in six WHO regions, enabling comparisons across diverse geographic and economic settings. The BMI–waist framework allowed identification of confirmed excess adiposity and may capture adiposity patterns not detected by BMI alone. In the analysis, random-effects meta-analysis accounted for between-country heterogeneity, multilevel models addressed country-level clustering in global analyses, and survey-weighted models generated region-specific estimates that are more representative of the underlying populations.

Despite its strengths, this study has several limitations. First, the cross-sectional design precludes causal inference about the associations between excess adiposity and cardiometabolic risk markers. Second, WHO STEPS do not include direct measures of body fat, such as DXA, CT, or MRI. Waist circumference and WHtR are practical population-level indicators of central adiposity, but they remain indirect measures. Third, WHO STEPS do not comprehensively assess organ dysfunction, symptoms, functional limitation, or full clinical history. Therefore, this study estimated anthropometrically confirmed excess adiposity rather than diagnosing clinical or preclinical obesity. Fourth, biochemical markers, including fasting glucose and cholesterol, were not consistently available across all countries, which may affect comparability and reduce the sample size for some outcome analyses. Point-of-care devices can differ from laboratory methods under field conditions, which may introduce some measurement variability across surveys. Fifth, some variables, including physical activity, diet, sedentary behaviour, and medication use, were self-reported and may be affected by recall or social desirability bias. Sixth, some regional estimates were based on small subgroup sizes, including those with no formal schooling in the Region of the Americas (N = 75). Participants aged 15–17 years accounted for 4.5% of the total sample and were not represented in the European or American regions, limiting the generalisability of estimates for this age group. Seventh, WHO STEPS do not include all countries, including some high-income countries, which might limit the generalisability of our findings to higher-resource settings. Finally, despite adjustment for major confounders, unmeasured factors, including genetic, early-life, and environmental influences, may have contributed to the observed associations.

This multi-country cross-sectional study, guided by the recent Lancet Commission framework, shows that BMI alone is insufficient for assessing excess adiposity and cardiometabolic risk in adolescents and young adults. Nearly one-third of the study population met the criteria for confirmed excess adiposity. Importantly, one in six participants had waist-confirmed adiposity despite not meeting BMI criteria for obesity, and this group also showed higher cardiometabolic risk burden. The burden varied across WHO regions and country income groups, highlighting the need for more complete approaches to adiposity assessment.

Incorporating low-cost, waist-based measures alongside BMI surveillance may help identify young people at higher cardiometabolic risk earlier. These findings could guide prevention efforts that promote physical activity and raise awareness of central adiposity as an early indicator of risk, although this study cannot determine how effective such strategies would be at lowering cardiometabolic risk. Strengthening adiposity assessment approaches in population surveillance may support broader efforts to address the growing burden of obesity and related cardiometabolic conditions among young people.

## Contributors

SBA, KYA, and VI conceptualised the study. SBA obtained and analysed the data, interpreted the results, drafted the original manuscript, and critically revised it. KYA, AGR, ST, and VI contributed to interpreting the findings and provided critical revisions to the intellectual content. SBA and KYA directly accessed and verified the underlying data reported in the manuscript. All authors read and approved the final manuscript.

## Data sharing statement

This study is a secondary analysis of the WHO STEPwise approach to NCD risk factor surveillance (STEPS) survey data. The datasets are available upon reasonable request from the WHO NCD Microdata Repository (https://extranet.who.int/ncdsmicrodata/index.php/home), subject to WHO data access and use policies.

## Declaration of interests

The Authors declare no conflict of interest.
