# Supplementary material for: Excess adiposity and early cardiometabolic risk markers among adolescents and young adults in 70 countries: a multi-country cross-sectional study
Source: eClinicalMedicine. 2026 Oct 27;101:104282. doi: 10.1016/j.eclinm.2026.104282 (PMC13735159; doi:10.1016/j.eclinm.2026.104282)
Supplement: Supplementary file 1 [file mmc1.docx]

**Supplementary material**

**Supplementary Methods**

**Overview**

WHO STEPwise Approach to Noncommunicable Disease Risk-Factor Surveillance (STEPS) is a standardised framework for monitoring major behavioural and metabolic risk factors for noncommunicable diseases (NCDs). The framework provides a common core of information across surveys while allowing countries to include additional items according to national priorities and resources.

**Target Population and Sampling**

The target population and sampling design are defined for each country-specific STEPS survey. The framework commonly targets adults aged 18–69 years, although countries may use different age ranges according to national priorities. Sampling frames and procedures may also vary between countries. STEPS surveys commonly use probability-based, multistage sampling, with geographic or population-based sampling units selected at successive stages before households and eligible participants are selected. The specific sampling stages, stratification variables, cluster definitions, and participant-selection procedures are determined by the design of each country-specific survey.

Sample sizes are determined according to factors including anticipated prevalence, desired precision and confidence level, design effect, and anticipated non-response. Consequently, sampling characteristics may differ across the country-year surveys included in the present analysis.

**Within-Household Participant Selection**

Within sampled households, eligible household members were identified according to the eligibility criteria specified for the respective STEPS survey, and one eligible participant was selected using the survey's prescribed random-selection procedure. Thus, participation was not based on selecting the household head by default, nor were all eligible household members routinely interviewed. The eligibility criteria, including the applicable age range, were defined for each country-specific survey before fieldwork. Consequently, the target age range could vary across country-year surveys. These country-specific differences were taken into account when harmonising the pooled dataset.

**Questionnaire and Measurements**

The STEPS Instrument comprises three main components. Step 1 collects information through an interview, including sociodemographic characteristics, tobacco and alcohol use, dietary behaviours, physical activity, and self-reported history of selected NCDs and risk factors. Step 2 involves physical measurements, including blood pressure, height, weight, and waist circumference, with additional measurements included where applicable. Step 3 comprises biochemical measurements, such as blood glucose and blood lipids, and may include other assessments depending on the country-specific implementation. Not all countries implement all three steps, and the availability of individual variables can therefore differ across surveys.

**Standardisation and Data Harmonisation**

The use of a common STEPS framework, standardised questionnaires, measurement procedures, training materials, and data-collection protocols supports comparability across participating surveys. Nevertheless, differences in sampling design, target population, implementation, measurement procedures, and availability of optional or expanded items may contribute to heterogeneity across country-year surveys.

For the present analysis, variables were harmonised using predefined definitions. Biochemical measurements recorded in different units were converted to common units using standard conversion factors, as described in the Methods.

**Survey Weights and Design Specification**

For the statistical analyses, the available survey sampling weights and country-year-specific stratification information were incorporated into the survey design. The anthropometric sampling weight (sampling weight 2) was used for analyses involving adiposity and hypertension, whereas the biochemical sampling weight (sampling weight 3) was used for analyses involving diabetes and elevated total cholesterol. The weights were rescaled within each country-year survey so that their sum corresponded to the analytic sample size for that survey. This rescaling changes the scale of the weights but preserves their relative weighting within each survey. Primary sampling unit (PSU) identifiers were not consistently available across the harmonised dataset. Consequently, the survey design was specified with individual observations treated as independent PSUs (ids = ~1). This allowed the available sampling weights and stratification information to be incorporated but did not account for within-PSU correlation arising from the original multistage sampling designs. Standard errors may therefore differ from those obtained using the complete original survey designs. This limitation was considered when interpreting the results.

**Table S1. Anthropometric Cut-offs Used to Define Confirmed Excess Adiposity**

| **Population** | **Sex** | BMI (kg/m²) | Waist Circumference (WC) (cm) | Waist-to-Height Ratio (WHtR) | Criteria |
| --- | --- | --- | --- | --- | --- |
| **Asian** | **Female** | ≥ 37.5 | — | — | Extreme BMI alone |
|  |  | 27.5 – 37.4 | ≥ 80 | — | BMI + WC |
|  |  | 27.5 – 37.4 | — | ≥ 0.5 | BMI + WHtR |
|  |  | — | ≥ 80 | ≥ 0.5 | WC + WHtR |
|  | **Male** | ≥ 37.5 | — | — | Extreme BMI alone |
|  |  | 27.5 – 37.4 | ≥ 90 | — | BMI + WC |
|  |  | 27.5 – 37.4 | — | ≥ 0.5 | BMI + WHtR |
|  |  | — | ≥ 90 | ≥ 0.5 | WC + WHtR |
| **Non-Asian** | **Female** | ≥ 40 | — | — | Extreme BMI alone |
|  |  | 30 – 39.9 | ≥ 80 | — | BMI + WC |
|  |  | 30 – 39.9 | — | ≥ 0.5 | BMI + WHtR |
|  |  | — | ≥ 80 | ≥ 0.5 | WC + WHtR |
|  | **Male** | ≥ 40 | — | — | Extreme BMI alone |
|  |  | 30 – 39.9 | ≥ 94 | — | BMI + WC |
|  |  | 30 – 39.9 | — | ≥ 0.5 | BMI + WHtR |
|  |  | — | ≥ 94 | ≥ 0.5 | WC + WHtR |

**Note:** Extreme BMI thresholds indicate excess adiposity regardless of waist circumference (WC) or waist-to-height ratio (WHtR). sex, and ethnicity-specific cut-offs were applied to account for variations in body composition and cardiometabolic risk[1].

**Table S2. Plausibility thresholds used to exclude implausible values**

| **Variable** | **Excluded values** |
| --- | --- |
| Height | <1 m or >2.7 m |
| Weight | <20 kg or >350 kg |
| Waist circumference | <0.2 m or >2.0 m |
| Body mass index (BMI)[2] | <10 kg/m² or >75 kg/m² |
| Waist-to-height ratio[2] | <0.2 or >2.0 |
| Systolic blood pressure[3] | <70 mmHg or >270 mmHg |
| Diastolic blood pressure[3] | <30 mmHg or >150 mmHg |
| Total cholesterol[4] | <67 mg/dL or >773 mg/dL |
| Fasting plasma glucose[5] | <30 mg/dL or >540 mg/dL |

Notes: Thresholds were applied during data cleaning to remove implausible values and were not used to define analytical outcomes. Cut-offs were aligned with prior multinational analyses and plausibility checks used in WHO STEPS-based and global pooled studies[6, 7].

**Table S3. Definitions and coding of explanatory variables**

| **Variable** | **Definition/coding** |
| --- | --- |
| **Sociodemographic factors** |  |
| Age group | 15–17 years; 18–24 years; 25–29 years |
| Sex | Male; Female |
| **Socioeconomic factors** |  |
| Education level | Secondary or more; no formal schooling; primary or less |
| Occupation | Working; not working |
| Country income group | Low-income; lower-middle-income; upper-middle-income; high-income |
| **Lifestyle factors** |  |
| Physical activity | Active (≥600 MET-min/week); inactive (<600 MET-min/week) |
| High sedentary time | No (<8 h/day); yes (≥8 h/day) |
| Fruit and vegetable intake | Adequate (≥5 servings/day); inadequate (<5 servings/day) |
| Current smoking | Current smoker; non-smoker (former smoker or never smoker) |
| Alcohol consumption | Non-drinker (0 drinks/week); drinker (>0 drinks/week) |
| Processed foods high in salt | Infrequent consumption; regular consumption |

**Note: Educational level** was harmonised across country surveys using the International Standard Classification of Education[8] informed approach, with country-specific education categories mapped into three common categories: no formal schooling, primary or less, and secondary or more. This approach was used to improve comparability across different education systems and category structures while maintaining adequate sample sizes within categories. **Country income groups** were based on the World Bank classification for the corresponding survey year[9]. **Physical activity** was assessed using WHO GPAQ, capturing work-related, transport-related, and leisure-time activities. Duration and intensity were converted into MET-minutes/week (vigorous = 8.0, moderate = 4.0). Participants were classified as physically active (≥600 MET-minutes/week) or physically inactive (<600 MET-minutes/week) according to the WHO guidelines[10].

**Smoking status** was classified as current smoker versus non-smoker based on self-reported current smoking status. Participants reporting current smoking were classified as smokers, while former smokers and never-smokers were grouped as non-smokers. **Alcohol consumption** was assessed using the self-reported number of standard drinks consumed during the previous 7 days. Participants were classified into two categories: non-drinker (0 drinks/week) and drinker (>0 drinks/week) [11]. **Fruit and vegetable intake** was categorised according to WHO recommendations as low intake (<5 servings/day) and adequate intake (≥5 servings/day)[12]. **Consumption of processed foods high in salt** was self-reported using WHO STEPS response categories and, for analysis, collapsed into infrequent and regular consumption.

**Figure S1. Participant flow diagram for the analytic sample**

Note: This flow diagram shows the derivation of the analytic sample from participants with valid anthropometric measurements, through to the final samples used for hypertension, diabetes, cholesterol, and composite cardiometabolic risk marker outcomes.

**Table S4: Weighted sample size and prevalence of exposure and outcomes, by country**

|  | **Confirmed excess adiposity** | | | | **Hypertension** | | | | **Diabetes** | | | | **High total cholesterol** | | | |
| --- | --- | --- | --- | --- | --- | --- | --- | --- | --- | --- | --- | --- | --- | --- | --- | --- |
| **Country** | **N** | **Missing, n (%)** | **No** | **Yes** | **N** | **Missing, n (%)** | **No** | **Yes** | **N** | **Missing, n (%)** | **No** | **Yes** | **N** | **Missing, n (%)** | **No** | **Yes** |
| Afghanistan(2018) | 1,317 | 0 (0.0%) | 53.2% | 46.8% | 1,307 | 4 (0.3%) | 78.5% | 21.5% | 1,173 | 44 (3.6%) | 93.5% | 6.5% | 1,223 | 0 (0.0%) | 85.9% | 14.1% |
| Algeria(2016) | 1,408 | 0 (0.0%) | 58.0% | 42.0% | 1,388 | 20 (1.4%) | 92.0% | 8.0% | 1,225 | 79 (6.0%) | 96.6% | 3.4% | 1,287 | 20 (1.5%) | 88.6% | 11.4% |
| Armenia(2016) | 423 | 0 (0.0%) | 64.7% | 35.3% | 393 | 29 (6.9%) | 89.1% | 10.9% | 341 | 94 (22.2%) | 97.1% | 2.9% | 343 | 91 (21.5%) | 89.9% | 10.1% |
| Aruba(2023) | 297 | 0 (0.0%) | 46.8% | 53.2% | 291 | 5 (1.7%) | 94.8% | 5.2% | 233 | 0 (0.0%) | 98.6% | 1.4% | 233 | 0 (0.0%) | 88.7% | 11.3% |
| Azerbaijan(2017) | 460 | 0 (0.0%) | 68.0% | 32.0% | 446 | 18 (3.9%) | 90.5% | 9.5% | 428 | 8 (1.8%) | 97.3% | 2.7% | 438 | 0 (0.0%) | 84.0% | 16.0% |
| Bahamas(2011) | 215 | 0 (0.0%) | 59.3% | 40.7% | 215 | 0 (0.0%) | 76.4% | 23.6% | - | - | - | - | - | - | - | - |
| Bangladesh(2018) | 1,842 | 0 (0.0%) | 80.5% | 19.5% | 1,838 | 5 (0.3%) | 91.4% | 8.6% | 1,489 | 38 (2.5%) | 96.9% | 3.1% | 1,486 | 39 (2.6%) | 82.3% | 17.7% |
| Belarus(2016) | 673 | 0 (0.0%) | 80.4% | 19.6% | 673 | 0 (0.0%) | 87.3% | 12.7% | 642 | 36 (5.3%) | 98.9% | 1.1% | 648 | 30 (4.5%) | 86.2% | 13.8% |
| Benin(2015) | 1,480 | 0 (0.0%) | 80.8% | 19.2% | 1,477 | 5 (0.3%) | 80.8% | 19.2% | 1,467 | 8 (0.5%) | 96.4% | 3.6% | 1,448 | 20 (1.4%) | 83.5% | 16.5% |
| Bolivia(2019) | 1,041 | 0 (0.0%) | 54.4% | 45.6% | 1,037 | 3 (0.3%) | 91.7% | 8.3% | - | - | - | - | - | - | - | - |
| Botswana(2014) | 1,450 | 0 (0.0%) | 81.0% | 19.0% | 1,446 | 4 (0.3%) | 81.8% | 18.2% | 1,256 | 202 (13.9%) | 98.9% | 1.1% | 1,245 | 216 (14.9%) | 95.5% | 4.5% |
| Brunei(2015) | 493 | 0 (0.0%) | 57.3% | 42.7% | 490 | 4 (0.8%) | 90.9% | 9.1% | 476 | 15 (3.1%) | 96.5% | 3.5% | 478 | 14 (2.9%) | 63.4% | 36.6% |
| Burkina Faso(2021) | 1,265 | 0 (0.0%) | 83.1% | 16.9% | 1,263 | 2 (0.2%) | 90.0% | 10.0% | 1,163 | 85 (6.8%) | 94.5% | 5.5% | 1,171 | 77 (6.1%) | 96.2% | 3.8% |
| Cabo Verde(2020) | 1,072 | 0 (0.0%) | 85.7% | 14.3% | 1,068 | 3 (0.3%) | 85.0% | 15.0% | 559 | 2 (0.4%) | 99.1% | 0.9% | 550 | 10 (1.8%) | 94.5% | 5.5% |
| Cambodia(2010) | 768 | 0 (0.0%) | 91.9% | 8.1% | 768 | 0 (0.0%) | 96.9% | 3.1% | 722 | 46 (6.0%) | 99.1% | 0.9% | 736 | 30 (3.9%) | 87.4% | 12.6% |
| Cayman Islands(2012) | 99 | 0 (0.0%) | 52.1% | 47.9% | 99 | 0 (0.0%) | 93.3% | 6.7% | - | - | - | - | - | - | - | - |
| Comoros(2011) | 993 | 0 (0.0%) | 68.5% | 31.5% | 991 | 3 (0.3%) | 87.2% | 12.8% | 411 | 557 (56.1%) | 98.5% | 1.5% | 214 | 756 (76.1%) | 84.3% | 15.7% |
| Cook Islands(2013) | 192 | 0 (0.0%) | 22.5% | 77.5% | 190 | 4 (2.1%) | 86.3% | 13.7% | 162 | 30 (15.6%) | 89.3% | 10.7% | 179 | 12 (6.2%) | 69.5% | 30.5% |
| Ecuador(2018) | 1,143 | 0 (0.0%) | 62.4% | 37.6% | 1,137 | 4 (0.3%) | 94.8% | 5.2% | 1,002 | 81 (7.5%) | 99.3% | 0.7% | 1,017 | 70 (6.5%) | 86.2% | 13.8% |
| Eritrea(2010) | 943 | 0 (0.0%) | 85.1% | 14.9% | 942 | 1 (0.1%) | 95.3% | 4.7% | 857 | 94 (10.0%) | 98.3% | 1.7% | 786 | 170 (18.0%) | 79.4% | 20.6% |
| Eswatini(2014) | 1,265 | 0 (0.0%) | 75.9% | 24.1% | 1,235 | 34 (2.7%) | 86.9% | 13.1% | 1,020 | 264 (20.9%) | 98.3% | 1.7% | 1,057 | 231 (18.3%) | 94.7% | 5.3% |
| Ethiopia(2015) | 3,671 | 0 (0.0%) | 93.1% | 6.9% | 3,649 | 21 (0.6%) | 89.0% | 11.0% | 3,292 | 384 (10.5%) | 98.5% | 1.5% | 3,347 | 330 (9.0%) | 97.0% | 3.0% |
| Fiji(2011) | 269 | 0 (0.0%) | 54.1% | 45.9% | 265 | 5 (1.9%) | 90.6% | 9.4% | 267 | 2 (0.7%) | 97.9% | 2.1% | - | 269 (100.0%) | - | - |
| French Polynesia(2010) | 906 | 0 (0.0%) | 45.7% | 54.3% | 570 | 357 (39.4%) | 88.3% | 11.7% | 542 | 387 (42.7%) | 99.1% | 0.9% | 542 | 380 (41.9%) | 68.4% | 31.6% |
| Gambia(2010) | 904 | 0 (0.0%) | 83.1% | 16.9% | 900 | 5 (0.6%) | 84.2% | 15.8% | - | - | - | - | - | - | - | - |
| Georgia(2016) | 550 | 0 (0.0%) | 65.5% | 34.5% | 542 | 7 (1.3%) | 88.0% | 12.0% | 356 | 12 (3.3%) | 99.8% | 0.2% | 364 | 4 (1.1%) | 90.4% | 9.6% |
| Grenada(2010) | 168 | 0 (0.0%) | 66.4% | 33.6% | 167 | 1 (0.6%) | 85.1% | 14.9% | - | - | - | - | - | - | - | - |
| Guyana(2016) | 714 | 0 (0.0%) | 58.1% | 41.9% | 704 | 9 (1.3%) | 88.9% | 11.1% | 229 | 97 (29.9%) | 96.8% | 3.2% | 238 | 84 (25.9%) | 72.4% | 27.6% |
| Iraq(2015) | 951 | 0 (0.0%) | 50.4% | 49.6% | 942 | 11 (1.2%) | 83.9% | 16.1% | 907 | 49 (5.2%) | 93.1% | 6.9% | 893 | 59 (6.2%) | 73.5% | 26.5% |
| Jordan(2019) | 1,344 | 0 (0.0%) | 66.3% | 33.7% | 1,337 | 8 (0.6%) | 95.3% | 4.7% | 727 | 30 (3.9%) | 97.3% | 2.7% | 761 | 10 (1.3%) | 92.3% | 7.7% |
| Kenya(2015) | 1,371 | 0 (0.0%) | 81.5% | 18.5% | 1,368 | 3 (0.2%) | 86.8% | 13.2% | 1,273 | 4 (0.3%) | 99.5% | 0.5% | 1,272 | 5 (0.4%) | 94.7% | 5.3% |
| Kiribati(2015) | 386 | 0 (0.0%) | 45.2% | 54.8% | 385 | 2 (0.5%) | 84.2% | 15.8% | 364 | 28 (7.3%) | 96.8% | 3.2% | 362 | 35 (9.1%) | 86.6% | 13.4% |
| Kuwait(2014) | 1,096 | 0 (0.0%) | 47.3% | 52.7% | 895 | 202 (18.4%) | 89.9% | 10.1% | 733 | 26 (3.4%) | 94.3% | 5.7% | 759 | 2 (0.3%) | 56.8% | 43.2% |
| Kyrgyzstan(2013) | 355 | 0 (0.0%) | 73.4% | 26.6% | 354 | 1 (0.3%) | 77.3% | 22.7% | 350 | 6 (1.7%) | 99.6% | 0.4% | 351 | 5 (1.4%) | 88.8% | 11.2% |
| Lao PDR(2013) | 637 | 0 (0.0%) | 85.6% | 14.4% | 635 | 2 (0.3%) | 92.4% | 7.6% | 620 | 15 (2.4%) | 99.4% | 0.6% | 623 | 12 (1.9%) | 88.1% | 11.9% |
| Lebanon(2017) | 227 | 0 (0.0%) | 39.6% | 60.4% | 223 | 3 (1.3%) | 81.1% | 18.9% | 111 | 6 (5.1%) | 97.0% | 3.0% | 114 | 2 (1.7%) | 51.1% | 48.9% |
| Lesotho(2012) | 378 | 0 (0.0%) | 74.1% | 25.9% | 376 | 1 (0.3%) | 83.1% | 16.9% | 330 | 55 (14.6%) | 98.7% | 1.3% | 330 | 53 (14.0%) | 97.0% | 3.0% |
| Liberia(2022) | 1,210 | 0 (0.0%) | 73.3% | 26.7% | 1,209 | 1 (0.1%) | 83.0% | 17.0% | 1,073 | 24 (2.2%) | 98.4% | 1.6% | 1,108 | 1 (0.1%) | 80.6% | 19.4% |
| Malawi(2017) | 1,255 | 0 (0.0%) | 86.7% | 13.3% | 1,251 | 5 (0.4%) | 88.3% | 11.7% | 1,106 | 19 (1.7%) | 98.8% | 1.2% | 1,116 | 10 (0.9%) | 95.9% | 4.1% |
| Moldova(2021) | 308 | 0 (0.0%) | 78.1% | 21.9% | 306 | 2 (0.6%) | 89.0% | 11.0% | 241 | 1 (0.4%) | 97.4% | 2.6% | 242 | 0 (0.0%) | 87.7% | 12.3% |
| Mongolia(2019) | 1,408 | 0 (0.0%) | 71.4% | 28.6% | 1,406 | 3 (0.2%) | 92.9% | 7.1% | 1,308 | 90 (6.4%) | 95.0% | 5.0% | 1,370 | 34 (2.4%) | 83.8% | 16.2% |
| Morocco(2017) | 981 | 0 (0.0%) | 59.8% | 40.2% | 980 | 2 (0.2%) | 90.1% | 9.9% | 816 | 25 (3.0%) | 96.0% | 4.0% | 816 | 31 (3.7%) | 95.8% | 4.2% |
| Myanmar(2014) | 734 | 0 (0.0%) | 86.0% | 14.0% | 729 | 2 (0.3%) | 90.4% | 9.6% | 713 | 18 (2.5%) | 98.7% | 1.3% | 700 | 27 (3.7%) | 83.2% | 16.8% |
| Nauru(2015) | 376 | 0 (0.0%) | 26.7% | 73.3% | 373 | 3 (0.8%) | 88.9% | 11.1% | 349 | 27 (7.2%) | 94.2% | 5.8% | 359 | 17 (4.5%) | 88.4% | 11.6% |
| Nepal(2019) | 1,411 | 0 (0.0%) | 79.5% | 20.5% | 1,409 | 3 (0.2%) | 86.5% | 13.5% | 1,301 | 35 (2.6%) | 97.5% | 2.5% | 1,338 | 0 (0.0%) | 94.7% | 5.3% |
| Niue(2011) | 240 | 0 (0.0%) | 42.2% | 57.8% | 239 | 1 (0.4%) | 91.8% | 8.2% | 227 | 13 (5.4%) | 94.4% | 5.6% | 201 | 39 (16.2%) | 87.5% | 12.5% |
| Qatar(2012) | 638 | 0 (0.0%) | 58.0% | 42.0% | 611 | 26 (4.1%) | 92.9% | 7.1% | 344 | 286 (44.8%) | 93.4% | 6.6% | 356 | 279 (43.7%) | 91.4% | 8.6% |
| Rwanda(2012) | 2,529 | 0 (0.0%) | 85.8% | 14.2% | 2,516 | 11 (0.4%) | 91.1% | 8.9% | 2,309 | 212 (8.4%) | 98.9% | 1.1% | 2,476 | 55 (2.2%) | 97.9% | 2.1% |
| Saint Lucia(2019) | 484 | 0 (0.0%) | 60.6% | 39.4% | 481 | 3 (0.6%) | 91.9% | 8.1% | - | - | - | - | - | - | - | - |
| Samoa(2013) | 458 | 0 (0.0%) | 43.3% | 56.7% | 454 | 5 (1.1%) | 91.9% | 8.1% | 439 | 15 (3.3%) | 87.3% | 12.7% | 445 | 9 (2.0%) | 94.3% | 5.7% |
| Sao Tome and Principe(2019) | 726 | 0 (0.0%) | 67.9% | 32.1% | 725 | 1 (0.1%) | 91.2% | 8.8% | 606 | 22 (3.5%) | 94.0% | 6.0% | 573 | 58 (9.2%) | 14.2% | 85.8% |
| Solomon Islands(2015) | 395 | 0 (0.0%) | 67.1% | 32.9% | 394 | 1 (0.3%) | 89.5% | 10.5% | 357 | 44 (11.1%) | 96.6% | 3.4% | 360 | 41 (10.4%) | 78.9% | 21.1% |
| Sri Lanka(2021) | 854 | 0 (0.0%) | 67.7% | 32.3% | 851 | 4 (0.5%) | 90.8% | 9.2% | 659 | 0 (0.0%) | 98.1% | 1.9% | 659 | 0 (0.0%) | 67.6% | 32.4% |
| Sudan(2016) | 2,146 | 0 (0.0%) | 80.1% | 19.9% | 2,145 | 2 (0.1%) | 79.5% | 20.5% | 1,875 | 116 (5.8%) | 97.3% | 2.7% | 1,991 | 18 (0.9%) | 92.6% | 7.4% |
| Syria(2017) | 510 | 0 (0.0%) | 43.4% | 56.6% | 506 | 3 (0.6%) | 83.2% | 16.8% | 230 | 20 (8.1%) | 92.7% | 7.3% | 239 | 7 (2.8%) | 71.0% | 29.0% |
| Tajikistan(2016) | 685 | 0 (0.0%) | 78.5% | 21.5% | 684 | 1 (0.1%) | 78.0% | 22.0% | 650 | 40 (5.8%) | 98.7% | 1.3% | 667 | 19 (2.8%) | 94.3% | 5.7% |
| Tanzania (Mainland)(2012) | 893 | 0 (0.0%) | 73.2% | 26.8% | 891 | 3 (0.3%) | 87.1% | 12.9% | 747 | 166 (18.6%) | 98.8% | 1.2% | 264 | 665 (74.5%) | 86.6% | 13.4% |
| Tanzania (Zanzibar)(2011) | 465 | 0 (0.0%) | 77.5% | 22.5% | 465 | 0 (0.0%) | 84.1% | 15.9% | 424 | 40 (8.6%) | 99.5% | 0.5% | 387 | 80 (17.2%) | 84.1% | 15.9% |
| Timor-Leste(2014) | 617 | 0 (0.0%) | 91.0% | 9.0% | 616 | 1 (0.2%) | 60.3% | 39.7% | 596 | 35 (5.7%) | 99.2% | 0.8% | 605 | 16 (2.6%) | 91.8% | 8.2% |
| Togo(2010) | 1,737 | 0 (0.0%) | 85.0% | 15.0% | 1,632 | 97 (5.6%) | 90.2% | 9.8% | 1,431 | 283 (16.3%) | 99.0% | 1.0% | 569 | 1,176 (67.7%) | 92.7% | 7.3% |
| Tokelau(2014) | 171 | 0 (0.0%) | 30.1% | 69.9% | 170 | 1 (0.6%) | 93.6% | 6.4% | 162 | 9 (5.3%) | 94.5% | 5.5% | 167 | 4 (2.3%) | 67.0% | 33.0% |
| Tonga(2017) | 798 | 0 (0.0%) | 22.7% | 77.3% | 784 | 13 (1.6%) | 82.6% | 17.4% | - | 798 (100.0%) | - | - | 791 | 7 (0.9%) | 85.5% | 14.5% |
| Turkmenistan(2018) | 952 | 0 (0.0%) | 65.8% | 34.2% | 952 | 0 (0.0%) | 90.5% | 9.5% | 924 | 29 (3.0%) | 97.5% | 2.5% | 938 | 16 (1.7%) | 84.7% | 15.3% |
| Tuvalu(2015) | 266 | 0 (0.0%) | 44.3% | 55.7% | 266 | 1 (0.4%) | 79.3% | 20.7% | 255 | 9 (3.4%) | 99.3% | 0.7% | 257 | 7 (2.6%) | 91.3% | 8.7% |
| Uganda(2014) | 1,422 | 0 (0.0%) | 85.6% | 14.4% | 1,417 | 6 (0.4%) | 84.4% | 15.6% | 1,305 | 18 (1.4%) | 99.7% | 0.3% | 1,322 | 0 (0.0%) | 95.9% | 4.1% |
| Ukraine(2019) | 550 | 0 (0.0%) | 77.6% | 22.4% | 542 | 20 (3.6%) | 88.9% | 11.1% | 337 | 185 (33.6%) | 96.4% | 3.6% | 353 | 173 (31.5%) | 78.4% | 21.6% |
| Vanuatu(2011) | 641 | 0 (0.0%) | 71.6% | 28.4% | 638 | 2 (0.3%) | 83.9% | 16.1% | 630 | 9 (1.4%) | 93.8% | 6.2% | 595 | 42 (6.6%) | 71.5% | 28.5% |
| Vietnam(2021) | 404 | 0 (0.0%) | 86.5% | 13.5% | 404 | 0 (0.0%) | 93.6% | 6.4% | 394 | 3 (0.8%) | 95.7% | 4.3% | 395 | 2 (0.5%) | 71.7% | 28.3% |
| Wallis and Futuna(2019) | 146 | 0 (0.0%) | 28.7% | 71.3% | 144 | 1 (0.7%) | 91.1% | 8.9% | 143 | 2 (1.4%) | 98.3% | 1.7% | - | 146 (100.0%) | - | - |
| Zambia(2017) | 1,458 | 0 (0.0%) | 82.9% | 17.1% | 1,438 | 15 (1.0%) | 87.7% | 12.3% | 1,219 | 235 (16.1%) | 96.6% | 3.4% | 1,319 | 144 (9.9%) | 96.1% | 3.9% |
| Total | 60,634 | 0 (0.0%) | 72.3% | 27.7% | 59,634 | 1,025 (1.7%) | 87.6% | 12.4% | 47,897 | 5,612 (10.5%) | 97.3% | 2.7% | 47,473 | 6,159 (11.5%) | 87.1% | 12.9% |

Confirmed excess adiposity and hypertension are reported among the anthropometric sample; diabetes and elevated total cholesterol are reported among the biochemical sample. "-" indicates no data available for that country. Country-specific prevalence for confirmed excess adiposity reflects weighted proportions calculated directly from each country's survey data; these may differ slightly from the corresponding estimates shown in Figure 1, which reflect random-effects meta-analytic estimates incorporating a variance-stabilising transformation.

**Table S5. Age- and sex-stratified determinants of confirmed excess adiposity**

|  | | **Age-stratified models** | | | **Sex-stratified models** | |
| --- | --- | --- | --- | --- | --- | --- |
| **Variable** | **Category** | **15-17 years** | **18-24 years** | **25-29 years** | **Males** | **Females** |
| Sex | Females (ref: Males) | 5.03 (3.56-7.11) | 3.87 (3.60-4.16) | 4.22 (3.92-4.55) | - | - |
| Age group | 18-24 years (ref: 15-17 years) | - | - | - | 1.35 (0.97-1.87) | 1.82 (1.53-2.18) |
|  | 25-29 years (ref: 15-17 years) | - | - | - | 2.74 (1.96-3.82) | 3.48 (2.91-4.17) |
| Education | No formal schooling (ref: Secondary+) | 0.78 (0.33-1.84) | 1.40 (1.21-1.61) | 0.90 (0.80-1.01) | 0.96 (0.79-1.16) | 1.00 (0.90-1.12) |
|  | Primary or less (ref: Secondary+) | 0.81 (0.56-1.18) | 1.04 (0.96-1.13) | 0.96 (0.88-1.04) | 0.79 (0.72-0.87) | 1.06 (0.99-1.14) |
| Occupation | Not working (ref: Working) | 1.89 (1.17-3.05) | 1.13 (1.05-1.22) | 1.04 (0.97-1.12) | 0.78 (0.71-0.87) | 1.18 (1.11-1.27) |
| Country income group | Lower-middle income (ref: Low-income) | 2.15 (0.77-5.99) | 1.23 (0.67-2.24) | 1.32 (0.76-2.30) | 2.59 (1.17-5.73) | 1.12 (0.64-1.95) |
|  | Upper-middle income (ref: Low-income) | 0.59 (0.18-2.01) | 1.99 (1.08-3.70) | 2.94 (1.60-5.40) | 8.70 (3.69-20.51) | 1.85 (1.01-3.40) |
|  | High-income (ref: Low-income) | 20.7 (3.07-139) | 5.31 (2.81-10.1) | 6.67 (3.57-12.5) | 18.06 (7.55-43.17) | 4.30 (2.29-8.05) |
| Physical activity | Inactive (ref: Active) | 1.57 (0.95-2.59) | 1.13 (1.04-1.23) | 1.31 (1.20-1.44) | 1.52 (1.36-1.69) | 1.12 (1.04-1.21) |
| High sedentary time | Yes (ref: No) | 0.82 (0.46-1.46) | 1.05 (0.95-1.17) | 1.09 (0.98-1.22) | 1.06 (0.94-1.20) | 1.05 (0.95-1.15) |
| Smoking | Yes (ref: No) | 1.66 (0.61-4.54) | 1.24 (1.13-1.37) | 0.81 (0.74-0.90) | 0.95 (0.87-1.04) | 0.91 (0.80-1.03) |

Adjusted odds ratios (OR) with 95% confidence intervals from separate survey-weighted mixed-effects logistic regression models, stratified by age group and by sex, each with a random intercept for country. Reference categories are shown in parentheses. Both sets of models were adjusted for education, occupation, country income group, physical activity, high sedentary time, and smoking, plus sex (age-stratified models) or age group (sex-stratified models) as applicable. "–" indicates the variable was not included as a predictor in that stratified model.

**Table S6: Weighted prevalence of cardiometabolic risk markers, by age group**

|  | **15-17 years** | | **18-24 years** | | **25-29 years** | |
| --- | --- | --- | --- | --- | --- | --- |
| **Marker** | **N** | **% (95% CI)** | **N** | **% (95% CI)** | **N** | **% (95% CI)** |
| Hypertension | 2112 | 6.3% (5.0-7.6%) | 27589 | 11.3% (10.4-12.2%) | 29908 | 14.0% (13.5-14.6%) |
| Diabetes | 1910 | 2.0% (1.2-2.8%) | 22419 | 2.5% (2.2-2.8%) | 23601 | 2.9% (2.6-3.2%) |
| Elevated total cholesterol | 1775 | 4.2% (3.1-5.3%) | 22776 | 11.1% (10.5-11.8%) | 22832 | 15.8% (15.2-16.5%) |

**Table S7: Individual cardiometabolic risk marker prevalence by BMI-waist adiposity category**

| **Adiposity category** | **Hypertension** | | **Diabetes** | | **Elevated total cholesterol** | |
| --- | --- | --- | --- | --- | --- | --- |
|  | **No** | **Yes** | **No** | **Yes** | **No** | **Yes** |
| No adiposity evidence | 29,305 (89.8%) | 3,338 (10.2%) | 29,389 (98.0%) | 601 (2.0%) | 26,702 (91.4%) | 2,515 (8.6%) |
| One waist criterion only | 4,988 (86.4%) | 783 (13.6%) | 5,165 (97.8%) | 118 (2.2%) | 4,471 (84.8%) | 801 (15.2%) |
| BMI-only obesity | 237 (77.7%) | 68 (22.3%) | 276 (94.4%) | 16 (5.6%) | 213 (70.9%) | 88 (29.1%) |
| Waist-confirmed excess adiposity | 6,855 (85.9%) | 1,124 (14.1%) | 6,981 (96.5%) | 253 (3.5%) | 5,893 (81.8%) | 1,307 (18.2%) |
| BMI-plus-waist or very-high BMI adiposity | 4,614 (78.4%) | 1,271 (21.6%) | 4,753 (94.5%) | 279 (5.5%) | 4,029 (74.5%) | 1,383 (25.5%) |
| No confirmed excess adiposity | 34,530 (89.2%) | 4,188 (10.8%) | 34,829 (97.9%) | 735 (2.1%) | 31,386 (90.2%) | 3,403 (9.8%) |
| Confirmed excess adiposity | 11,469 (82.7%) | 2,395 (17.3%) | 11,734 (95.7%) | 533 (4.3%) | 9,923 (78.7%) | 2,690 (21.3%) |

Values are weighted n (%), row percentages, from the biochemical sample. The bottom two rows show the same markers as in the binary-confirmed excess adiposity classification.

**Figure S2. Prevalence of mutually exclusive BMI–waist adiposity classification categories.**

Note: Categories are mutually exclusive; each participant is classified into exactly one category. No adiposity evidence indicates no BMI-defined obesity or waist-based adiposity. BMI-only obesity indicates BMI-defined obesity without waist-based adiposity or very-high BMI. Waist-confirmed adiposity indicates both high WC and WHtR ≥0.50 without BMI-defined obesity. BMI-plus-waist or very-high BMI adiposity indicates very-high BMI, or BMI-defined obesity with at least one waist-based marker. Confirmed excess adiposity includes waist-confirmed adiposity and BMI-plus-waist or very-high BMI adiposity. BMI = body mass index

**References**

1.	Tan K: **Appropriate body-mass index for Asian populations and its implications for policy and intervention strategies**. *The lancet* 2004.

2.	Jelaković B, Baretić M, Dika Ž, Jelaković A, Josipović J, Kos J, Majer M, Mišigoj-Duraković M, Musil V, Musić Milanović S: **Worldwide trends in underweight and obesity from 1990 to 2022: a pooled analysis of 3663 population-representative studies with 222 million children, adolescents, and adults**. *The Lancet* 2023, **16**(403 (10431)):1027-1050.

3.	Zhou B, Carrillo-Larco R, Danaei G, Riley L, Paciorek C, Stevens G, Gregg E, Bennett J, Solomon B, Singleton R: **NCD Risk factor collaboration (NCD-RisC). Worldwide trends in hypertension prevalence and progress in treatment and control from 1990 to 2019: a pooled analysis of 1201 population-representative studies with 104 million participants**. *Lancet* 2021, **398**(10304):957-980.

4.	Topor-Madry R: **Repositioning of the global epicentre of non-optimal cholesterol**. *Nature* 2020.

5.	Smolen J, Burmester G, Combeet B: **NCD Risk Factor Collaboration (NCD-RisC). Worldwide trends in diabetes since 1980: a pooled analysis of 751 population-based studies with 4· 4 million participants. Lancet2016; 387: 1513–30—In this Article, Catherine Pelletier**. 2016.

6.	Ahmed KY, Aychiluhm SB, Thapa S, Tegegne TK, Ketema DB, Kassa ZY, Kibret GD, Duko B, Shifti DM, Bore MG: **Cardiometabolic outcomes among adults with abdominal obesity and normal body mass index**. *JAMA network open* 2025, **8**(10):e2537942-e2537942.

7.	Carrillo-Larco RM, Guzman-Vilca WC, Tarazona-Meza C, Xu X, Bernabe-Ortiz A: **Prevalence of pre-clinical and clinical obesity in adults: Pooled analysis of 56 population-based national health surveys**. *PLOS Global Public Health* 2025, **5**(7):e0004838.

8.	Unesco: **International standard classification of education (ISCED) 2011**: UNESCO; 2012.

9.	Bank W: **World Bank country and lending groups**. *World Bank* 2020.

10.	Armstrong T, Bull F: **Development of the world health organization global physical activity questionnaire (GPAQ)**. *Journal of Public Health* 2006, **14**(2):66-70.

11.	**Alcohol's Effects on Health** [<https://www.niaaa.nih.gov/alcohols-effects-health/alcohol-drinking-patterns>]

12.	Nations. WHOFaAOotU: **Diet, nutrition, and the prevention of chronic diseases: report of a joint WHO/FAO expert consultation**. In: *Technical Report Series.* vol. 916. Geneva: World Health Organization; 2003.
